# Analytical Validation of a Telomerase Reverse Transcriptase (*TERT*) Promoter Mutation Assay

**DOI:** 10.1210/clinem/dgae134

**Published:** 2024-03-05

**Authors:** Priyanka C Iyer, Ramona Dadu, Anna Barque, Cleslei Zanelli, Xingyu Zheng, Huimin Jiang, P Sean Walsh, Yangyang Hao, Jing Huang, Joshua P Klopper, Richard T Kloos, Maria Cabanillas

**Affiliations:** Department of Endocrine Neoplasia and Hormonal Disorders, Division of Internal Medicine, The University of Texas MD Anderson Cancer Center, Houston, TX 77030, USA; Department of Endocrine Neoplasia and Hormonal Disorders, Division of Internal Medicine, The University of Texas MD Anderson Cancer Center, Houston, TX 77030, USA; Veracyte Inc, South San Francisco, CA 94080, USA; Veracyte Inc, South San Francisco, CA 94080, USA; Veracyte Inc, South San Francisco, CA 94080, USA; Veracyte Inc, South San Francisco, CA 94080, USA; Veracyte Inc, South San Francisco, CA 94080, USA; Veracyte Inc, South San Francisco, CA 94080, USA; Veracyte Inc, South San Francisco, CA 94080, USA; Veracyte Inc, South San Francisco, CA 94080, USA; Veracyte Inc, South San Francisco, CA 94080, USA; Department of Endocrine Neoplasia and Hormonal Disorders, Division of Internal Medicine, The University of Texas MD Anderson Cancer Center, Houston, TX 77030, USA

**Keywords:** *TERT*, analytical validation, thyroid nodule, thyroid cancer, Afirma, assay

## Abstract

**Context:**

Telomerase reverse transcriptase (*TERT*) promoter-mutated thyroid cancers are associated with a decreased rate of disease-free and disease-specific survival. High-quality analytical validation of a diagnostic test promotes confidence in the results that inform clinical decision-making.

**Objective:**

This work aimed to demonstrate the analytical validation of the Afirma *TERT* promoter mutation assay.

**Methods:**

*TERT* promoter C228T and C250T variant detection in genomic DNA (gDNA) was analyzed by assessing variable DNA input and the limit of detection (LOD) of variant allele frequency (VAF). The negative and positive percentage agreement (NPA and PPA) of the Afirma *TERT* test was examined against a reference primer pair as was the analytical specificity from potential interfering substances (RNA and blood gDNA). Further, the intrarun, interrun, and interlaboratory reproducibility of the assay were tested.

**Results:**

The Afirma *TERT* test is tolerant to variation in DNA input amount (7-13 ng) and can detect expected positive *TERT* promoter variants down to 5% VAF LOD at 7 ng DNA input with greater than 95% sensitivity. Both NPA and PPA were 100% against the reference primer pair. The test remains accurate in the presence of 20% RNA or 80% blood gDNA for an average patient sample that typically has 30% VAF. The test also demonstrated a 100% confirmation rate when compared with an external next-generation sequencing–based reference assay executed in a non-Veracyte laboratory.

**Conclusion:**

The analytical robustness and reproducibility of the Afirma *TERT* test support its routine clinical use among thyroid nodules with indeterminate cytology that are Afirma Genomic Sequencing Classifier suspicious or among Bethesda V/VI nodules.

Thyroid nodules are commonly detected in clinical practice, and the majority are benign (1). When thyroid nodule fine-needle aspiration (FNA) cytology is indeterminate, molecular testing is often used to help distinguish benign from malignant thyroid nodules (2). Additionally, prognostic information, based on molecular variants and fusions detected, may inform a patient's tumor behavior, risk of recurrence, and optimal initial therapy (3-5). The Afirma Genomic Sequencing Classifier (GSC) uses next-generation RNA sequencing and whole-exome analysis combined with machine-learning algorithms to provide a benign or suspicious result in nodules with indeterminate thyroid nodules (6). The primary goal of the test is to allow patients to avoid unnecessary surgery by ruling out thyroid cancer. In thyroid nodules with Bethesda V and VI cytology, Afirma shifts from primarily a diagnostic test to one of prognostication. The Afirma Xpression Atlas (XA) component of the GSC provides detailed information about expressed molecular variants and fusions (7). Afirma XA identifies molecular alterations in 593 genes, reporting on 905 variants and 235 fusions. Identification of genomic variants and fusions in Bethesda V and Bethesda VI nodules may provide clinically valuable information about thyroid cancers’ pretreatment extent, prognosis, and future chemotherapeutic targets (8-10).

In 2013, telomerase reverse transcriptase promoter mutations (*TERT*), C228T and C250T, were identified in thyroid cancer as mutually exclusive but independently powerful in thyroid oncogenesis (11). Prevalence of C228T and C250T has been reported as 9.7% and 2.1% in papillary thyroid cancer (PTC), 15.7% and 2.5% in follicular thyroid cancer (FTC), 33.8% and 15.0% in poorly differentiated thyroid cancer (PDTC), and 37.7% and 4.1% in anaplastic thyroid cancer (ATC), respectively (12). Comparatively, the relative distribution was found to be dominant for *TERT* C228T among the 2 mutations: 82.5%, 86.2%, 69.2%, and 90.2% in PTC, FTC, PDTC, and ATC, respectively. The *TERT* promoter mutations were identified with tumors that behave aggressively such as PDTC and ATC, consistent with other studies (13). Prognostically, several studies have shown the coexistence of *BRAF*V600E and *TERT* promoter mutations to have higher-risk clinicopathologic features in PTC than either mutation independently, such as extrathyroidal extension, regional lymph node metastases, and distant metastases (14-16). Furthermore, coexistence of *BRAF*V600E and *TERT* or *RAS* and *TERT* mutations has been shown to be associated with higher recurrence rate and mortality both in PTC and FTC, while these variants independently have a modest effect (17). Finally, cancer-specific survival was lower in patients with *TERT* promoter mutations in encapsulated angioinvasive FTC and widely invasive FTC compared to those without (18).

Thus, there is a compelling need for a diagnostic test to determine the existence of the *TERT* promoter mutations early in the identification of malignant thyroid nodules to inform management decisions that can affect clinical outcomes of these patients. The purpose of this study is to show the analytical validation of the Afirma *TERT* promoter mutation assay, demonstrating its accurate, consistent, and reproducible results.

## Materials and Methods

### Specimens

This study was conducted under an institutional review board exemption for Veracyte's protocol to use leftover clinical specimens (Copernicus Group IRB). Prospective FNA clinical samples were acquired and shipped under controlled temperature and stored at −80 °C until extraction. A description of blood samples used in this study has been previously described (11).

### DNA Extraction, AmpliSeq Library Preparation, and Sequencing

Genomic DNA (gDNA) from clinical FNA specimens was extracted following the AllPrep Micro Kit procedure (QIAGEN). Extracted gDNA yields were determined using Qubit 1× dsDNA High Sensitivity and Qubit Flex Fluorometer (Invitrogen). A total of 7 to 13 ng of DNA were processed using the AmpliSeq for Illumina Custom Panel (Illumina) on the Microlab STARlet robotics platform (Hamilton) as per the manufacturer's guidelines. No template (Low TE), and negative and positive controls manufactured with a mixture of cell lines’ gDNA (gDNA) harboring the 2 *TERT* variants analyzed in this test (C228T [c.-124C > T] and C250T [c.-146C > T]) were included in each library preparatory batch. Libraries were then normalized, pooled, and sequenced on MiniSeq System instruments (Illumina).

### Bioinformatics Pipeline

**Figure 1.**
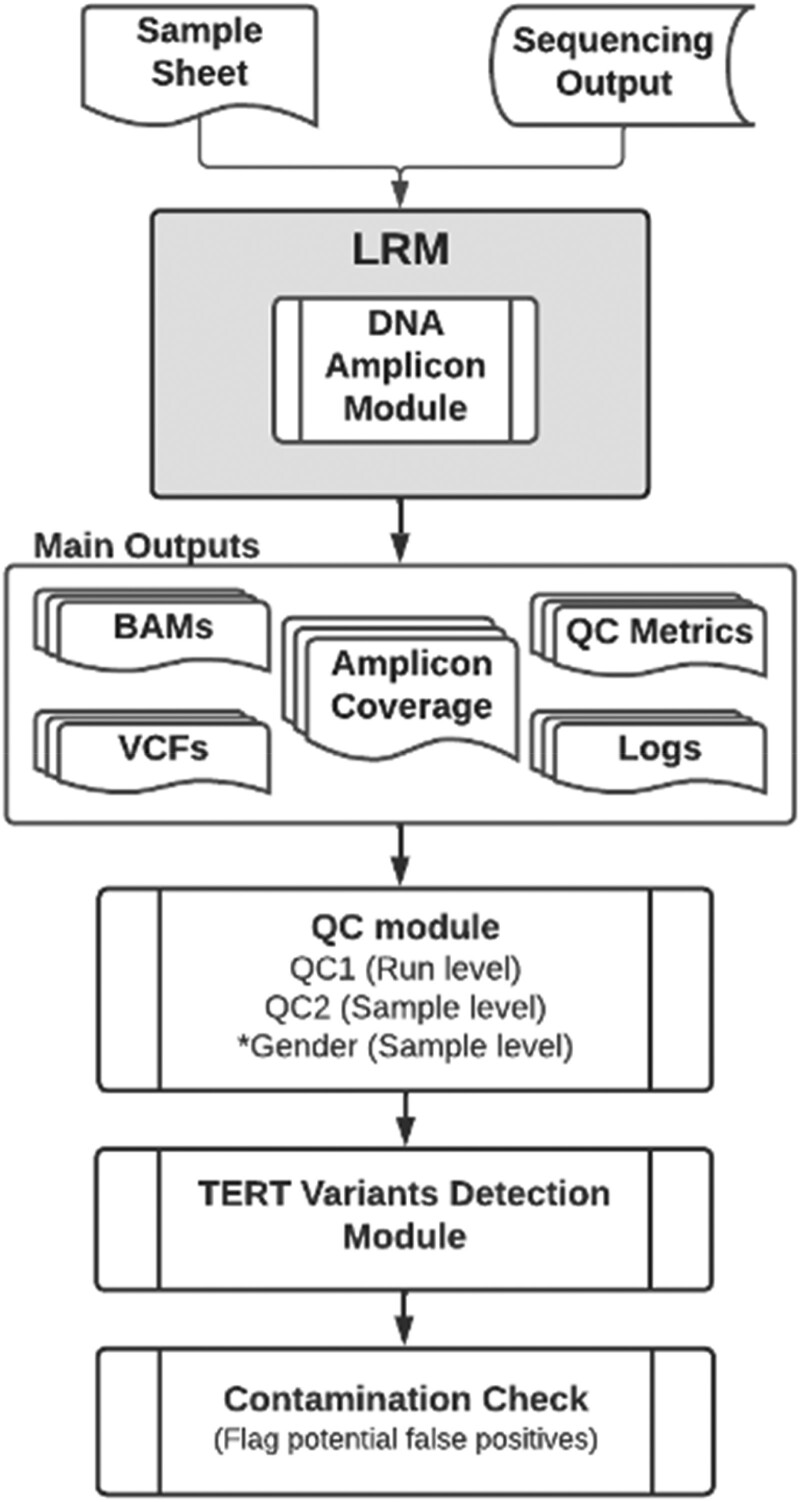
TERT bioinformatics pipeline overview (refer to Supplement 1 (https://doi.org/10.6084/m9.figshare.25013885.v1) for details). *Gender: this refers to the DNA AmpliSeq-derived sex.

Sequencing data from the *TERT* DNA AmpliSeq assay underwent processing through the bioinformatics pipeline, primarily using the Illumina Local Run Manager (LRM, version 2.4.1.2636) and DNA Amplicon module (version 2.1.0.19). Fig. 1 illustrates the bioinformatics pipeline. The DNA Amplicon module serves the purpose of demultiplexing, mapping sequencing reads to the reference genome hg19, and storing the results in standard BAM format. Additionally, it generates quality control metrics, logs, amplicon coverage information, and performs variant calling, storing the variants in standard variant cell format (VCF) format. Further details of the bioinformatics pipeline can be found in Supplement 1 (https://doi.org/10.6084/m9.figshare.25013885.v1) (19).

## Results

Laboratory-to-laboratory accuracy was assessed by testing 90 samples in 2 different Veracyte laboratories (the research and development laboratory and the Clinical Laboratory Improvement Amendments laboratory), 48 of which were positive for the *TERT* C228T variant, 12 for the *TERT* C250T variant, and 30 had no detectable *TERT* C228T or C250T variants, as determined in the R&D laboratory using a *TERT* primer pair different from the commercial *TERT* test. The specimen panel was selected to cover a representative range of variant allele frequency (VAF) expected from the patient stream, and final histology of these lesions was unknown. Following sequencing, analysis was performed to identify variants. As summarized in Table 1, 100% concordance between laboratories was observed among all *TERT*-positive and -negative instances.

**Table 1.** Results of the accuracy study

		Veracyte laboratory 1			
		No. of instances			
		Variant not detected	C228T positive	C250T positive	Total
Veracyte Laboratory 2	Variant not detected	120	0	0	120
	C228T positive	0	48	0	48
	C250T positive	0	0	12	12
	Total	120	48	12	180
	Concordance	100%	100%	100%	

**Figure 2.**
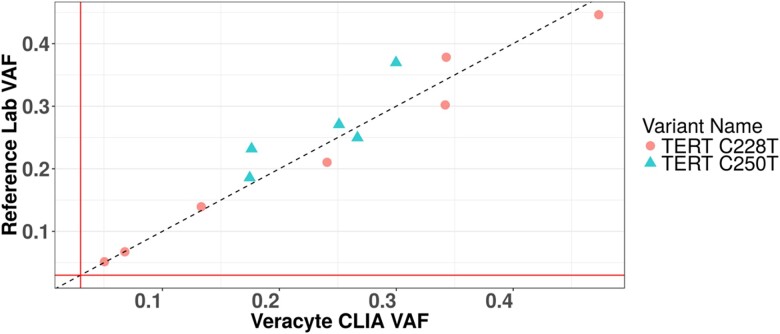
External confirmation. The reference laboratory uses a capture-based next-generation sequencing assay interrogating 638 genes. In 12 patient samples, 100% of C228T and C250T *TERT* promoter mutations were detected and correlated with the Veracyte assay.

Variant calls made using the *TERT* test were confirmed by comparison with data from the OncoDeep test (OncoDNA Lab) using the same DNA samples derived from patient FNA specimens. The OncoDeep test is an enrichment-based next-generation sequencing assay that amplifies and analyses 638 genes including the *TERT* gene. Samples were selected to cover a broad VAF range. One sample was from a lesion with Bethesda III cytology (C228 at 5.1% VAF), and the rest were from lesions with Bethesda V or VI cytology. Final histology of these tumors is unknown in the laboratory. Twelve patient samples positive for *TERT* promoter mutations with a range of *TERT* VAF (5.0%-46.2% by the *TERT* test) were analyzed. *TERT* AmpliSeq data from the 12 FNA samples were compared with the OncoDeep assay data, and 100% concordance was observed for C228T and C250T *TERT* variants (Fig. 2) with a VAF Pearson correlation of 0.96 and a Spearman correlation of 0.98.

FNA samples from 7 patient samples, each positive for at least 1 of the 2 nucleotide variants, and 3 patient samples with no detectable *TERT* variants were tested in triplicate in a single run to determine intrarun variability. Interrun variation was determined by testing the same patient samples across 4 runs in which reagent lots, operators, and equipment were varied. The *TERT* test demonstrated 100% intraplate and interplate reproducibility for the 2 variants in all samples. No unexpected variants were detected for any of the samples both in intraplate and interplate replicates (Table 2).

**Table 2.** Results for intrarun and interrun precision

No. of samples: 10		
Intrarun reproducibility (3 replicates/sample)	Positive call concordance (expected variants detected)	100% (95% CI, 83.9%-100%)
	Negative call concordance (C228T and C250T variants not detected)	100% (95% CI, 91.0%-100%)
Interrun reproducibility (4 runs/sample)	Positive call concordance (expected variants detected)	100% (95% CI, 95.7%-100%)
	Negative call concordance (C228T and C250T variants not detected)	100% (95% CI, 97.7%-100%)

**Figure 3.**
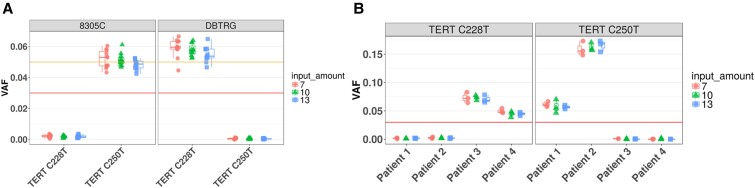
A, Four *TERT*-positive patient samples, 5% to 17.5% variant allele frequency (VAF), 3 replicates at each input level (7 ng, 10 ng, 13 ng). B, Two *TERT*-positive cell line genomic DNAs diluted to 5% VAF, 11 replicates at each input level (7 ng, 10 ng, 13 ng); 10 ng is the nominal input.

The analytical sensitivity of the *TERT* test was tested by varying the input DNA amount on both *TERT*-positive cell lines and patient samples. Two cell lines, each containing 1 of the 2 *TERT* variants analyzed in this test—DBTRG-05MG containing the C228T variant and 8305C containing the C250T variant—were blended with gDNA from the NA12878 cell line to reach VAF of 5%. Eleven replicates at each of the gDNA input levels of 7.0, 10.0, and 13.0 ng were tested. The expected variants at 5% VAF were detected in 100% of the replicates at all 3 DNA input levels. Four patient samples were also tested in triplicate at gDNA input levels of 7.0, 10.0, and 13.0 ng. A total of 100% of patient sample replicates had their expected variants detected at all 3 DNA input levels tested. Therefore, the limit of detection for TERT C228T or C250T variants at 5% VAF is 7 ng for gDNA input (Fig. 3).

**Figure 4.**
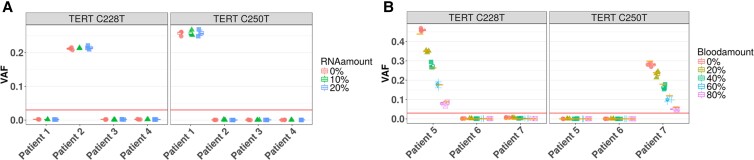
A, *TERT*-variant detection status of all patient sample replicates at 10% RNA and 20% RNA contamination are concordant with expectations. B, *TERT*-variant detection status of all replicates at all added blood levels (0%, 20%, 40%, 60%, 80%) are concordant with expectations for patient samples with 30% or greater variant allele frequency (VAF).

Total RNA was identified as one potential interfering substance. Small amounts of total RNA may be found in the gDNA extracted from patient FNAs due to the nucleic acid extraction process. Studies performed in R&D showed that less than 10% of RNA may be present in the DNA eluate when loading the DNA columns with pure RNA (UHR, Thermo Fisher Scientific, QS0639) in lysis buffer (data not shown). This may be the worst-case scenario since normally the lysate will contain both gDNA and RNA, and gDNA will be preferentially bound to the silica membrane. In this study, the effect of total RNA as a potentially interfering substance was tested at levels of 0% (baseline), 10%, and 20% of the total gDNA mass. Samples were created by mixing total RNA at 10% and 20% of the amount of DNA from the 4 FNA samples obtained from patient thyroid nodules, each containing either a defined variant (C228T or C250T), or with no detectable variants as shown in Fig. 4A (0% RNA). These samples were processed through the *TERT* test, along with the controls (gDNA samples with no added RNA). All samples were tested in triplicate. As shown in Fig. 4, TERT variant detection status was not affected by RNA amount up to 20%, the highest level that was tested.

Blood is a potential interfering substance arising from the thyroid FNA procedure, as traces of blood may be present in patient samples and the gDNA from blood cells can be coextracted with gDNA from thyroid tissues. The presence of blood gDNA coextracted with thyroid FNA gDNA is expected to dilute observed VAF, leading to possible false negatives. This study tested spiking-in 0% (baseline), 20%, 40%, 60%, and 80% blood gDNA extracted from a healthy donor into representative thyroid FNA gDNAs (Supplementary Fig. S1), harboring either a defined *TERT* variant (C228T at 44% VAF or C250T at 30% VAF; see Fig. 4B at 0% blood) or with no detectable variants, to fully assess the effect on *TERT* promoter variant detection status (https://doi.org/10.6084/m9.figshare.25013885.v1) (19). All samples were tested in triplicate along with the assay controls. The *TERT* variant detection status of all patient samples was as expected and 100% concordant at all blood amount levels tested (see Fig. 4B). Therefore, the *TERT* test can tolerate up to 80% of blood gDNA, the highest level tested, for an average patient sample of at least 30% VAF. The observed blood content in patient samples was estimated, based on 637 blood gene markers, to be no more than 80% for 95% of 5000 random patient samples.

## Discussion

The Afirma GSC uses whole-exome next-generation RNA sequencing analysis combined with machine-learning algorithms to create molecular classifiers and report on genomic variants and fusions to improve the diagnostic accuracy of thyroid nodules with Bethesda III and IV cytology and provide prognostic information in those nodules as well as those with Bethesda V and VI cytology (6, 20).

Telomeres are condensed DNA protein structures on the ends of chromosomes, protecting chromosomes from fusions and DNA damage (21). With each cell division, telomeres shorten, allowing DNA damage and decreased cellular proliferation. Increased telomerase activity, as can occur with *TERT* promoter mutations, can maintain telomere length and induce cell immortality, which may mechanistically explain the generally more aggressive clinicopathologic features associated with *TERT* promoter mutated thyroid carcinomas (22, 23). Identification of *TERT* promoter mutations requires a DNA-based assay as gene promoter sequences are not transcribed and would not be detected by the Afirma GSC assay (24). Therefore, a new Afirma DNA-based assay has been developed to detect *TERT* promoter mutations.

Analytical validation is an important aspect of characterizing a new test, as outlined by the Evaluation of Genomic Applications in Practice and Prevention (EGAPP) Working Group and the Centers for Disease Control's ACCE (Analytic and Clinical validity, Clinical utility and associated Ethical) Project (25, 26). All analytical validation studies were performed in a prospective manner, whereby the acceptance criteria for each study were determined (1) based on previously approved design requirements and (2) prior to the study being performed in the laboratory. Here we report the analytical validation of the Afirma *TERT* assay and demonstrate its accuracy, that is, 100% concordance of *TERT* detection between Veracyte internal laboratories as well as against an external reference laboratory, and its robustness to various technical variations along the entire process of library generation, sequencing, and algorithm analysis. Additionally, we show the tolerance of the test to the effect of potential interference substances including up to 20% RNA contamination and blood contamination up to 80% of the sample input.

To our knowledge, this is the first published analytical validation of a *TERT* promotor assay in a commercially available thyroid nodule molecular test. Studies showing the development and accuracy of diagnostics are important. A false-positive *TERT* result could lead to overtreatment, while a false-negative test may not alert clinicians to a worse prognosis and thyroid cancer outcome. Demonstrating test accuracy, durability of results in the presence of clinically expected contaminants, and reproducibility can reassure patients and clinicians of the validity of Afirma *TERT* promoter test results. As has been described, a positive *TERT* promoter mutation result provides important diagnostic and prognostic information for patients with thyroid nodules and thyroid cancer, though a limitation of this study is that those outcomes were not known for the clinical samples tested in this analytical validation work (27, 28). Future studies should include prospective analyses of thyroid cancer management changes related to *TERT*-positive thyroid nodules and cancer to see if a more aggressive initial approach to therapy improves thyroid tumor morbidity and mortality.

## Data Availability

Original data generated and analyzed during this study are included in this published article or in the data repositories listed in “References.”

## Abbreviations

ATC: anaplastic thyroid cancer
FNA: fine-needle aspiration
FTC: follicular thyroid cancer
gDNA: genomic DNA
GSC: Genomic Sequencing Classifier
LOD: limit of detection
NPA: negative percentage agreement
PDTC: poorly differentiated thyroid cancer
PPA: positive percentage agreement
PTC: papillary thyroid cancer
TERT: telomerase reverse transcriptase
VAF: variant allele frequency
